# Physiological and Psychological Responses to Three Distinct Exercise Training Regimens Performed in an Outdoor Setting: Acute and Delayed Response

**DOI:** 10.3390/jfmk6020044

**Published:** 2021-05-24

**Authors:** Stefano Benítez-Flores, Carlos A. Magallanes, Cristine Lima Alberton, Todd A. Astorino

**Affiliations:** 1 Department of Physical Education and Health, Higher Institute of Physical Education, University of the Republic, Montevideo 11600, Uruguay; camagallanes@gmail.com; 2 Neuromuscular Assessment Laboratory, Physical Education School, Federal University of Pelotas, Pelotas 625-96055630, Brazil; tinialberton@yahoo.com.br; 3 Department of Kinesiology, California State University San Marcos, San Marcos, CA 92096-0001, USA; astorino@csusm.edu

**Keywords:** high-intensity interval training, sprint interval training, continuous training, fatigue, recovery

## Abstract

The aim of this study was to compare the acute responses to three time-matched exercise regimens. Ten trained adults (age, maximum oxygen consumption (VO_2max_), and body mass index (BMI) = 25.9 ± 5.6 yr, 50.9 ± 5.4 mL·kg^−1^·min^−1^, and 22.1 ± 1.8 kg·m^−2^) completed sprint interval training (SIT) requiring 14 × 5 s efforts with 35 s of recovery, high-intensity interval training (HIIT) consisting of 18 × 15 s efforts at ~90% of peak heart rate (HR_peak_) with 15 s of recovery, and vigorous continuous training (CT) consisting of 8.75 min at ~85 %HR_peak_, in randomized order. Heart rate, blood lactate concentration, rating of perceived exertion, affective valence, and enjoyment were monitored. Moreover, indices of neuromuscular function, autonomic balance, diet, mental stress, incidental physical activity (PA), and sleep were measured 24 h after each session to analyze the magnitude of recovery. Both HIIT and CT exhibited a greater %HR_peak_ and time ≥ 90 %HR_peak_ than SIT (*p* < 0.05). Blood lactate and rating of perceived exertion were higher in response to SIT and HIIT vs. CT (*p* < 0.05); however, there were no differences in enjoyment (*p* > 0.05). No differences were exhibited in any variable assessed along 24 h post-exercise between conditions (*p* > 0.05). These data suggest that HIIT and CT accumulate the longest duration at near maximal intensities, which is considered a key factor to enhance VO_2max_.

## 1. Introduction

For many decades, the efficacy of interval training in human wellness has consistently been shown [1]. There are two distinct categories of interval training, consisting of high-intensity interval training (HIIT), which involves submaximal efforts near the workload associated with maximal heart rate or oxygen uptake, and sprint interval training (SIT), which involves supramaximal bouts requiring “all-out” effort [2]. Both training modalities induce similar physiological adaptations to continuous training (CT) with a lower training volume [1]. Despite the many well-documented benefits of HIIT and SIT on various indices of cardiometabolic health [1], many interval regimens require extremely demanding efforts that may elicit negative perceptions (e.g., Tabata protocol and Wingate-based SIT) [3,4] that could be impractical for non-athletic populations.

Since the level of self-efficacy, motivation, and enjoyment are predictors of physical activity participation (PA) [5], it has been suggested that reducing bout duration elicits more positive affective responses [4]. This is a great advantage of modified SIT which has been shown to significantly improve the cardiometabolic profile in healthy and unhealthy adults [2,6,7,8]. In addition, modified SIT requires lower total volume [6,8] which could mitigate the lack of time indicated as the main barrier to engaging in regular PA [5]. In fact, current guidelines recommend small blocks of vigorous PA given that they induce several health benefits [9]. However, most interval training regimens are conducted in laboratory environments using expensive technology [10] that limit the external validity of these findings to real-world settings such as the home or workplace where minimal equipment is available.

Chronic adaptations to exercise training are caused by the accumulation of acute cardiovascular and metabolic responses [11] which highlights the importance of assessing the change in variables including heart rate (HR) and blood lactate concentration to exercise. However, only a few studies have compared differences in these variables in response to interval training regimens performed outside of a laboratory. Warr-di Piero et al. [12] recruited a sample of heterogeneous athletes to perform four different regimens of HIIT with bout durations equal to 10, 50, 90, and 130 s. The results showed greater blood lactate and rating of perceived exertion in response to longer bouts (i.e., 90 and 130 s) compared to the shorter efforts. This result is in agreement with data reported by Cipryan et al. [13] acquired in a laboratory. In healthy subjects, Eigendorf et al. [14] evaluated physiological responses to SIT, HIIT, and CT having identical mean power output. This study showed no significant difference between conditions in oxygen consumption (VO_2_), respiratory exchange ratio, or plasma ammonia concentration. Nevertheless, the exercise duration was substantial (~75 min), reducing its applicability in untrained populations.

Besides the acute responses to exercise, latent physiological responses also influence training adaptations. In addition, the magnitude of delayed-onset muscle soreness (DOMS) in the days following exercise may impair adherence to PA, particularly in sedentary adults [15]. Nevertheless, a recent study by Farias-Junior et al. [16] in overweight men showed no alterations in numerous markers of muscle damage and inflammation 48 h after HIIT and CT matched for volume. Yet, to the best of our knowledge, there are no data concerning changes in variables including sleep, mental stress, or neuromuscular function in response to time-matched exercise regimens completed using accessible equipment in field conditions. At present, it has been suggested that heart rate variability (HRV), jump performance, and psychometric questionnaires are reliable and practical non-invasive measurements to elucidate the overall recovery status from prior exercise [17].

Thus, the aim of this study was to compare acute physiological and psychological responses to three different field-based exercise protocols with identical total duration yet different structure, and additionally, to observe changes in various markers of recovery for 24 h post-exercise employing affordable tools. Our hypothesis is that no differences in HR values and recovery status will be evident between conditions due to the similar exercise workload.

## 2. Materials and Methods

### 2.1. Subjects

Twelve healthy adults (range 20–40 yr; 8 males, 4 females) participated in the study during November/December 2019 (Table 1). The inclusion criteria were: (1) absence of musculoskeletal injuries and cardiometabolic risk factors; (2) highly physically active according to the short IPAQ; (3) not consuming any nutritional supplements, drugs, or tobacco products; (4) not competing professionally in any sport; and (5) previous experience in intense exercise training. Participants were instructed to abstain from PA and alcohol consumption for 48 h before all sessions and also to avoid stimulating drinks (e.g., coffee, mate, etc.) in the morning/afternoon of each session. In addition, they were asked not to change lifestyle habits (e.g., work, sleep, food, etc.) throughout the experiment. Prior to involvement, all procedures, potential risks, and benefits were fully explained to participants and subsequently, they provided their informed consent. This study was carried out in accordance with the principles stipulated in the Declaration of Helsinki of 1975, revised in 2013.

### 2.2. Study Design

**Figure 1:**
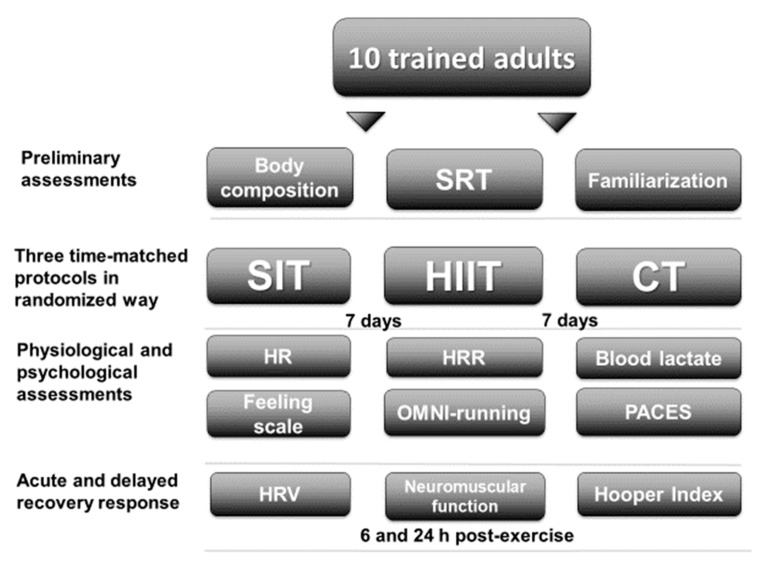
Study design. SRT = shuttle run test; SIT = sprint interval training with 5 s efforts; HIIT = high-intensity interval training with 15 s efforts; CT = continuous training; HR = heart rate; HRR = heart rate recovery; OMNI-running = OMNI-running rating of perceived exertion scale; PACES = physical activity enjoyment scale; HRV = heart rate variability.

This study adopted a randomized crossover design that consisted of one session to measure various physical and morphological variables followed by 3 time-matched sessions of endurance training (SIT, HIIT, and CT). The design was developed with the aim of being integrated in real-world circumstances, using low-cost tools that are easy to employ. All procedures took place on a 400 m outdoor public track. Every session consisted of different efforts (“all-out”, intermittent submaximal, and continuous submaximal) with a similar internal load (i.e., HR) and completion of ~9 min of exercise per session, and was separated by 7 days. All sessions were held on the same days (Monday and Tuesday), time of day (0800–1100 a.m. and 200–500 p.m.), and season (Spring), with similar environmental conditions (18–25 °C temperature, 40–50% humidity, and 11–20 km·h^−1^ wind). Dietary consumption was monitored for 24 h before the first exercise session using diet recalls, and we requested that participants replicate the same food intake 24 h before the other sessions. Physiological and psychological responses were monitored during all sessions. In addition, in the 24 h after each session, various indicators of residual neuromuscular/metabolic fatigue were monitored to determine the level of recovery/stress. Additionally, incidental PA and sleep were controlled as possible confounding variables (Figure 1).

### 2.3. Procedures

#### 2.3.1. Preliminary Assessments

In the first session, the participants completed the short IPAQ followed by anthropometric measurements. Subsequently, the 20-m shuttle run test (SRT) was performed to estimate VO_2max_. Finally, they were familiarized with the exercise protocols.

##### Body Composition

The following anthropometric measurements were recorded: height (cm), weight (kg), body fat (%), and body skeletal muscle mass (%), utilizing a digital body composition bioimpedance sensor HBF-514C, OMRON (Kyoto, Japan).

##### 20-m Shuttle Run Test

The SRT is a practical approach to assess cardiorespiratory fitness in non-laboratory settings without need for sophisticated equipment [18]. The test consists of running for as long as possible between 2 lines separated by 20 m with a rhythm imposed by audio. The initial speed was equal to 8.5 km·h^−1^ and was increased by 0.5 km·h^−1^ every minute. The speed obtained in the last stage is considered as the maximal aerobic speed. The end of the test is determined when the distance of 20 m cannot be covered in two consecutive efforts. The HR was monitored during the test using a telemetric system Firstbeat Technologies Ltd. (Jyväskylä, Finland). Peak heart rate (HR_peak_) was registered and VO_2max_ was estimated according to the formula proposed by Leger and Gadoury [18] (VO_2max_ = (6 × maximal aerobic speed) − 27.4).

##### Familiarization

Participants were familiarized with the psychological scales and neuromuscular function tests by completing 2–3 repetitions of each test. Additionally, they were familiarized with the three exercise protocols as they completed 1 min of CT at ~85% HR_max_ (estimated according to: 220 minus age), two to three 15 s bouts of HIIT at ~90% HR_max_ (same as CT), and two 5 s bouts of SIT.

##### Exercise Protocols

The warm-up included 3 min of running at a self-selected pace. Total time for every mode was ~14 min. The CT and HIIT bouts were prescribed at intensities equal to 85 and 90% of the HR_peak_. During each session, HR was instantaneously monitored and feedback was provided so that the subjects maintained the target intensity. Previously, our group identified that HR was equal to ~90% HR_max_ in response to a very similar modified SIT protocol as the one used in the current study [19]. During SIT, participants were instructed to run as fast as they could for 5 s. After every repetition of HIIT and SIT, passive pauses were used to facilitate recovery [11], and then participants were warned by a sound signal to run in the opposite direction. The characteristics of these protocols are described in Table 2.

#### 2.3.2. Physiological and Psychological Assessments in the Three Exercise Protocols

##### External and Internal Load

Throughout all exercise regimens, the distance achieved was quantified by placing landmarks every 5 m on the track, and an investigator individually followed each participant to record the distance run.

Internal load of the sessions was determined using HR data collected with chest straps Firstbeat Technologies Ltd. (Jyväskylä, Finland) that were later exported to the Firstbeat Sports software version 4.7.3.1, Firstbeat Technologies Ltd. (Jyväskylä, Finland). The intensity descriptors selected were maximal (HR_max_), mean (HR_mean)_, minimum (HR_min_), percent peak HR (%HR_peak_), time ≥ 70 %HR_peak_ (i.e., between 70 and 80% of the HR_peak_), time ≥ 80 %HR_peak_ (i.e., between 80 and 90% of the HR_peak_), and time ≥ 90 %HR_peak_ (i.e., between 90 and100% of the HR_peak_).

##### HR Recovery (HRR)

The relative HRR was defined as the difference between HR registered at the end of exercise (HR_end_) and after 30, 60, 90, and 120 s of recovery (i.e., HRΔ30s, HRΔ60s, HRΔ90s, and HRΔ120s). This variable was recorded as the subjects walked at ~4 km·h^−1^, similar to a previous study [20].

##### Blood Lactate Concentration

Fingertip blood samples (15 µL of blood) were collected pre- and 4 min post-exercise using disposable lancets and placed in reagent strips for subsequent analysis with a portable lactate analyzer Accutrend, Roche Diagnostics (Basel, Switzerland).

##### Affective Valence, Rating of Perceived Exertion, and Enjoyment

The Feeling scale was employed pre- and immediately post-exercise to measure the affective valence (pleasure and displeasure), ranging from −5 (very bad) to +5 (very good) [21]. To determine perceived exertion pre- and immediately post-exercise, the OMNI-running rating of perceived exertion scale (OMNI-running) was used [22]. This scale was validated for adults during walking/running exercise and is easier to interpret and apply than the classic Borg scale (6–20). The physical activity enjoyment scale (PACES) is traditionally used to characterize the level of enjoyment induced by exercise. In the present study, a valid and reliable Spanish short version was completed immediately post-exercise [23].

#### 2.3.3. Acute and Delayed Recovery Response

##### Heart Rate Variability (HRV)

To assess HRV, the participants were oriented comfortably in the supine position and requested to breathe normally and avoid any kind of movements throughout data acquisition. The subjects remained in this position for 2 min pre- and post-exercise, and 6 and 24 h post-exercise. For the analysis, Firstbeat Sports software version 4.7.3.1, Firstbeat Technologies Ltd. (Jyväskylä, Finland) was adopted, and only the second minute of recording was analyzed because the first minute is considered a stabilization period. This type of recording has been used to assess autonomic regulation accurately in a field environment [24]. The variable selected was the root mean square of successive differences between R-R intervals (RMSSD), established as the strongest indicator to monitor autonomic balance [17].

##### Neuromuscular Function

To observe the neuromuscular function, the PUSH band version 2.0 PUSH Inc. (Toronto, ON, Canada) was employed with waist belt secured properly. The PUSH is a portable device based on a triaxial accelerometer and gyroscope that register samples at 1000 Hz, transforming them into one 200 Hz signal. This device was created to track the velocity of movement during a variety of strength exercises and shows adequate validity and reliability [25]. Two tests were chosen to assess the impact of the different exercise regimens on neuromuscular function. First, the countermovement jump (CMJ) was performed, given that it is a validated technique to determine lower-body power [26]. Second, the reactive strength index (RSI) was evaluated through completion of 10 continuous rebound jumps to assess the capacity of reactive strength. The RSI 10/5 stiffness was considered, including only the best 5 jumps [27]. The CMJ and RSI tests were performed before, 4 min post-exercise, and 6 and 24 h post-exercise. In the case of the CMJ, the average of 2 repetitions separated by 1 min recovery was analyzed, while in the case of RSI, only 1 repetition was analyzed.

##### Hooper Index

The Hooper index is an ecological method to assess general parameters of recovery and wellness for athletes [28]. This psychometric questionnaire is based on ratings relative to fatigue, stress level, muscle soreness, and sleep quality, scored on a seven-point Likert scale with 1-point increments scores of 1–7, with 1 and 7 representing very, very low (very, very good in the case of sleep) and very, very high (very, very bad in the case of sleep), respectively. The Hooper index was administered pre-, immediately post-exercise, and 6 and 24 h post-exercise in all conditions.

##### Incidental PA

Incidental PA was monitored by accelerometry GT3X, ActiGraph, LCC. (Pensacola, FL, USA) 48 h before and 24 h after completion of each exercise condition. Subjects were instructed to wear the device on their non-dominant wrist, and to only remove it for any aquatic activity. Analysis days were included when ≥ 10 h·day of recording was attained. The daily energy expenditure (EE) and incidental PA patterns were calculated, applying an epoch of 1 s and a frequency of 100 Hz, with the Freedson’s algorithm for cutoff points: (1) light, <1951 counts·min; (2) moderate, 1952–5724 counts·min; (3) vigorous, 5725–9498 counts·min; and (4) very vigorous, >9499 counts·min [29]. All estimations were completed in manufacturer software ActiLife version 6.13.4, ActiGraph, LCC. (Pensacola, FL, USA).

##### Sleep

Sleep indicators were calculated 48 h before (i.e., 2 nights) and 24 h after (i.e., 1 night) for each condition, utilizing accelerometry GT3X, ActiGraph, LCC. (Pensacola, FL, USA) with an epoch of 60 s based on the Cole and Kripke algorithm [30]. All estimations were performed using manufacturer ActiLife version 6.13.4, ActiGraph, LCC. (Pensacola, FL, USA).

### 2.4. Statistical Analysis

Sample size was defined with the following input parameters in G*Power version 3.1.9.7, Düsseldorf University https://www.psychologie.hhu.de/arbeitsgruppen/allgemeine-psychologie-und-arbeitspsychologie/gpower (Düsseldorf, Germany): (1) F test for one group and three measurements; (2) effect size of 0.45; (3) a-value of 0.05; (4) statistical power of 0.80; and (5) correlation between measures of 0.5. The calculated sample size was 10.

Data are presented as mean ± SD. Normality was assessed by means of standard distribution measures, visual inspection of *Q*–*Q* plots, box plots, and the Shapiro–Wilk test. Variables with a non-normal distribution were log-transformed for analysis. Where normalization was not possible, non-parametric methods were used. One-way repeated measures ANOVA was conducted to examine differences in select variables (i.e., distance, HR, HRR, PACES, etc.) during the three sessions. In addition, this technique was used to compare changes in incidental PA and sleep (before 48 h and after 24 h). A series of 2 (time equal to pre- and post-) × 3 (condition equal to SIT, HIIT, and CT) two-way repeated measures ANOVA was performed to assess differences in blood lactate. Additionally, the same analysis with a 4 × 3 model was used to assess differences across time (pre-, post-, post-6 h, and post-24 h) and condition (SIT, HIIT and CT) for the evaluation of HRV and RSI. Mauchly’s sphericity was tested and if sphericity could not be assumed the Greenhouse–Geisser correction was used. When required, pairwise comparisons were conducted using Bonferroni’s corrections. In the case of non-normally distributed variables (OMNI-running, Feeling scale, CMJ, and Hooper index) an analysis with non-parametric techniques was applied using the Friedman rank sum test with post-hoc Nemenyi. Effect sizes were calculated using *n_p_^2^* in order to examine the magnitude of the differences between the three sessions. For non-parametric variables, the Kendall’s W coefficient (*k*) was used as the measure of the Friedman test effect size. Cohen’s *d* (for normal variables) and *r* = *z*/√*N* (for non-normal variables) were calculated for ES analyses representing ≤ 0.20 as a small effect, 0.50 as a medium effect, and ≥0.80 as a large effect. Parametric statistics were performed with IBM SPSS version 23.0 (Armonk, NY, USA) and non-parametric statistics with R (R Core Team, 2018). In all cases, the alpha level was set at *p* < 0.05. All graphics were made with GraphPad Prism version 6.01 (San Diego, CA, USA).

## 3. Results

One female participant withdrew from the study due to time constraints. Additionally, data from one female participant were not considered since in one session, the HR signal was not detected. All other participants were able to complete the three training conditions.

### 3.1. External and Internal Load

#### 3.1.1. HR

**Figure 2:**
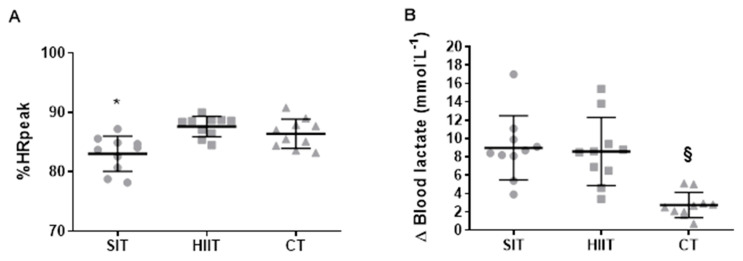
Individual response of (**A**) %HR_peak_ through the training protocols and individual (**B**) Δ of blood lactate through the training protocols. SIT = sprint interval training with 5 s efforts; HIIT = high-intensity interval training with 15 s efforts; CT = continuous training. * *p* < 0.05 differences vs. HIIT and CT. § *p* < 0.05 differences vs. HIIT and SIT.

The completed distance was significantly different (*p* < 0.001; *n_p_^2^* = 0.98) across all conditions (*p* < 0.001) (Table 2). Significant differences were detected in HR_max_ (*p* < 0.001; *n_p_^2^* = 0.57) as it was higher in HIIT vs. SIT (*p* = 0.050; *d* = 0.80) and CT (*p* = 0.027; *d* = 0.33). In addition, the results show that HR_mean_ and %HR_peak_ (*p* < 0.001; *n_p_^2^* = 0.60) were lower in SIT than HIIT (*p* = 0.003; *d* = 0.99) and CT (*p* = 0.029; *d* = 0.77) (Figure 2A), although there was no difference in the HR_min_ (*p* = 0.105; *n_p_^2^* = 0.22). For time ≥ 70 %HR_peak_, a significant difference was found (*p* = 0.032; *k* = 0.34) although post-hoc analyses showed no differences between means (*p* > 0.05). For time ≥ 80 %HR_peak_, no significant difference was shown (*p* = 0.196; *k* = 0.16) but for time ≥ 90 %HR_peak_, a significant difference between conditions occurred (*p* = 0.017; *k* = 0.40) as it was lower in SIT than HIIT (*p* = 0.011; *r* = 0.80) (Table 2).

#### 3.1.2. HRR and Blood Lactate

The results show a significant difference in HR_end_ (*p* ≤ 0.001; *n_p_^2^* = 0.57) that was higher in response to HIIT than SIT (*p* = 0.002; *d* = 1.26) and CT (*p* = 0.031; *d* = 0.88). A significant difference was observed in HRΔ90s (*p* = 0.002; *n_p_^2^* = 0.51) that was lower in SIT vs. HIIT (*p* = 0.031; *d* = 1.41) and CT (*p* = 0.002; *d* = 1.27). Moreover, a significant difference was identified in HRΔ120s (*p* = 0.007; *n_p_^2^* = 0.43) that was lower in response to SIT vs. HIIT (*p* = 0.018; *d* = 1.28). No differences were observed in HRΔ30s (*p* = 0.517; *n_p_^2^* = 0.10) or HRΔ60s (*p* = 0.070; *n_p_^2^* = 0.26) across conditions (Table 2).

Significant condition (*p* < 0.001; *n_p_*^2^ = 0.65), time (*p* < 0.001; *n_p_*^2^ = 0.92), and condition×time interaction (*p* < 0.001; *n_p_*^2^ = 0.65) effects were identified for blood lactate. Post-hoc outcomes revealed higher post-exercise values vs. pre- in SIT (*p* < 0.001; *d* = 3.71), HIIT (*p* < 0.001; *d* = 3.21), and CT (*p* < 0.001; *d* = 2.10). Additionally, higher post-exercise values occurred in SIT (11.5 ± 3.4 mmol L^−1^; *p* < 0.001; *d* = 2.26) and HIIT (10.6 ± 3.7 mmol L^−1^; *p* = 0.005; *d* = 1.80) than CT (5.4 ± 1.7 mmol L^−1^). These data are shown in Figure 2B.

#### 3.1.3. Affective Valence, Perceived Exertion, and Enjoyment

**Figure 3:**
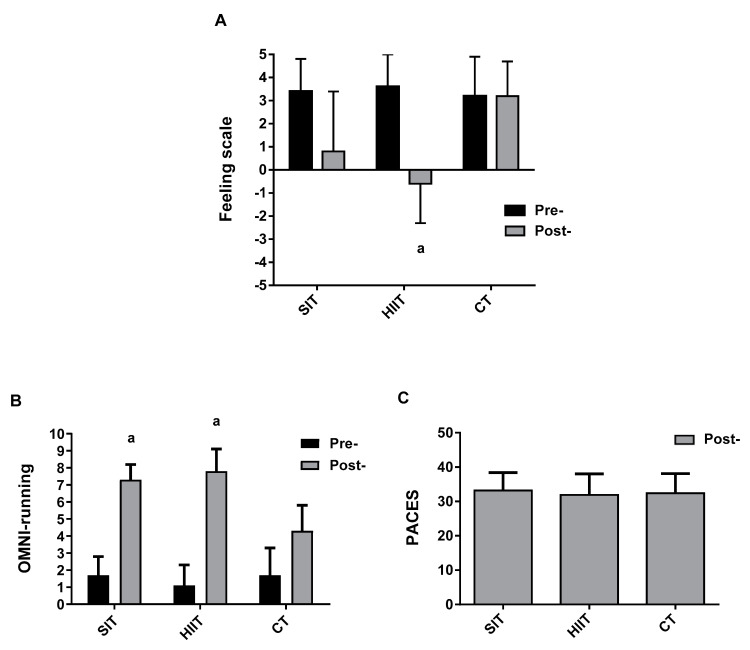
Group response for (**A**) Feeling scale; (**B**) OMNI-running, and (**C**) PACES through the training protocols. SIT = sprint interval training with 5 s efforts; HIIT = high-intensity interval training with 15 s efforts; CT = continuous training. ^a^ *p* < 0.05 differences vs. pre-training.

Significant main effects of condition (*p* = 0.030; *k* = 0.37), time (*p* = 0.020; *k* = 0.54), and a training×time interaction (*p* < 0.001; *k* = 0.54) were identified for the Feeling scale. Post-hoc analysis identified differences in affective valence during HIIT (*p* = 0.009; *r* = 0.89) and lower post-exercise values in HIIT versus CT (*p* = 0.020; *r* = 0.80) (Figure 3A).

There were significant main effects of condition (*p* = 0.007; *k* = 0.50), time (*p* = 0.002; *k* = 1), and training×time interaction (*p* < 0.001; *k* = 0.85), indicating a different pattern in response to the three regimens for OMNI-running. Pairwise comparisons showed differences in pre- vs. post- for SIT (*p* = 0.002; *r* = 0.89) and HIIT (*p* < 0.001; *r* = 0.88). No inter-conditions differences were found at any time (*p* > 0.05) (Figure 3B).

No differences in PACES were observed between conditions (*p* = 0.734; *n_p_^2^* = 0.034) (Figure 3C).

### 3.2. Acute and Delayed Recovery Response

#### 3.2.1. HRV

**Figure 4:**
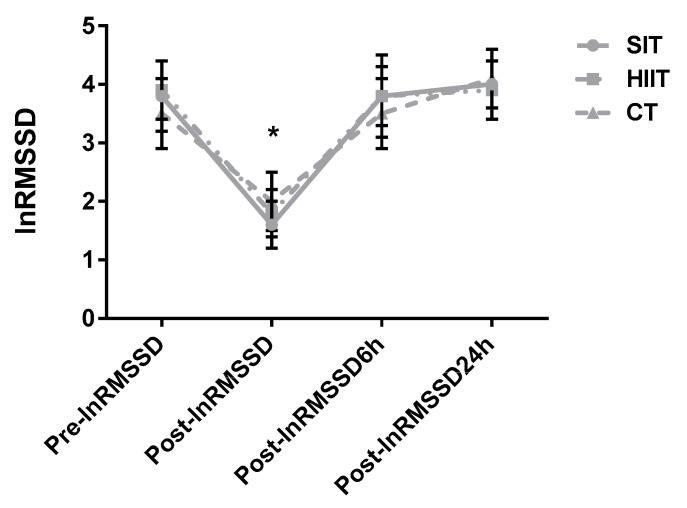
Group response comparisons of the lnRMSSD at the different time points. SIT = sprint interval training with 5 s efforts; HIIT = high-intensity interval training with 15 s efforts; CT = continuous training. * *p* < 0.05 differences vs. pre-training for all protocols.

There were significant main effects of time (*p* < 0.001; *n_p_^2^* = 0.91) and training×time (*p* = 0.011; *n_p_^2^* = 0.26) for lnRMSSD. Pairwise comparisons revealed decrements in post-exercise measures for SIT (*p* < 0.001; *d* = 4.31), HIIT (*p* < 0.001; *d* = 4.63), and CT (*p* < 0.001; *d* = 2.71) and lower values than the other time points (*p* < 0.01). No differences between conditions were found at any time point (*p* > 0.05) and there was no main effect of condition (*p* = 0.875; *n_p_^2^* = 0.01) (Figure 4).

#### 3.2.2. CMJ and RSI

**Figure 5:**
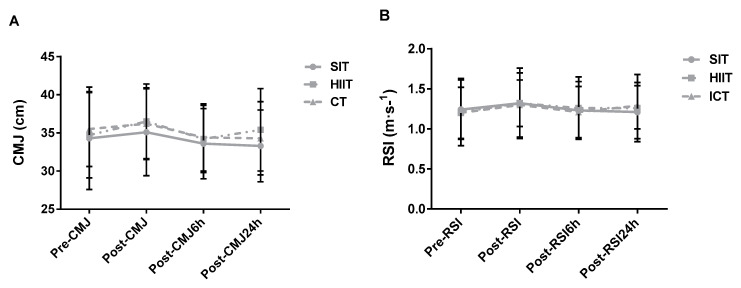
Group response comparisons of the (**A**) CMJ and (**B**) RSI at the different time points. SIT = sprint interval training with 5 s efforts; HIIT = high-intensity interval training with 15 s efforts; CT = continuous training.

Significant main effects of time (*p* = 0.002; *k* = 0.49) and training×time (*p* = 0.002; *k* = 0.26) interaction were noted, yet post-hoc tests showed no differences between means. No main effect of training was observed (*p* = 0.120; *k* = 0.21) (Figure 5A). The results show no main effects of training, time, or training×time interaction (*p* > 0.05; *n_p_^2^* ≥ 0.22) for RSI (Figure 5B).

#### 3.2.3. Hooper Index

Regarding fatigue, significant training (*p* = 0.007; *k* = 0.50), time (*p* < 0.001; *k* = 0.63), and training×time interaction (*p* < 0.001; *k* = 0.50) effects were identified. Paired comparisons showed a difference between pre- vs. post-exercise for SIT (*p* = 0.005; *r* = 0.89) and HIIT (*p* = 0.010; *r* = 0.84). Regarding stress, significant time (*p* = 0.020; *k* = 0.34) and training×time interaction (*p* = 0.004; *k* = 0.25) effects were detected, without a main effect of training (*p* = 0.140; *k* = 0.19). Neither paired differences were found (*p* > 0.05). Regarding muscle soreness, significant time (*p* = 0.002; *k* = 0.51) and training×time interaction (*p* = 0.002; *k* = 0.27) effects were observed, without a main effect of training (*p* = 0.070; *k* = 0.27). Neither paired differences were noted (*p* > 0.05). Regarding sleep quality, no effect was detected (*p* > 0.05; *k* ≥ 14).

#### 3.2.4. Incidental PA and sleep

No differences were detected before and after for all parameters of incidental PA (*p* > 0.05; *n_p_^2^* ≥ 0.26) or sleep (*p* > 0.05; *n_p_^2^* ≥ 0.024) (Table 3).

## 4. Discussion

This is the first study that compared acute and delayed responses of time-matched interval training regimens utilizing brief efforts vs. continuous exercise in real-world settings. Our results show no significant differences between HIIT and CT in HR_mean_ and time ≥ 90 %HR_peak_, yet these outcomes are lower in response to SIT. Second, blood lactate concentration and perception of effort were higher in SIT and HIIT than CT. Third, no differences between protocols were detected in acute fatigue and general recovery status over 24 h, suggesting that brief bouts of interval exercise or vigorous exercise do not interfere with subsequent recovery.

Data from many recent reviews demonstrate that low-volume interval training is effective to enhance cardiorespiratory fitness, glycemic regulation, and body fat [2,8]. However, to date there is still a lack of results elucidating responses to different interval training protocols in relation to CT, since most studies compare regimens having dissimilar exercise load [31]. Obtaining a better understanding of acute responses to these regimens is important, as different iterations of peak workload, bout number, intensity, duration, mean workload, and intensity and duration of recovery may elicit specific acute disturbances of homeostasis that in turn promote specific physiological adaptations [32].

It has been suggested that for exercise training to improve central and peripheral factors associated with O_2_ transport and utilization, participants should exercise at intensities near VO_2max_ and spend at least several minutes at this target intensity [11]. In elite cyclists, Almquist et al. [33] compared acute responses to brief (30 s) vs. long intervals (5 min) having the same volume and work:rest ratio. Their results show that brief intervals led to a 14% higher mean power output and 153% longer duration above 90% HR_peak_. Our data show that 15 s bouts of HIIT elicited the highest HR throughout the session in the form of a greater %HR_peak_ (+5%) and longest duration running at intensities ≥ 90 %HR_peak_ (+500%) (Figure 2 and Table 2). Furthermore, HIIT and CT revealed similar HR_min_ and HR_max_ values, suggesting that both sessions elicit the same cardiorespiratory intensity despite different running velocity. These findings coincide with the information presented by Tschakert et al. [34] and propose that reduced durations of effort have a greater ability to stabilize cardiac function.

During intense, brief efforts of exercise, including HIIT and SIT, the contribution of phosphagen and glycolytic metabolism is high. When recoveries are too brief and/or VO_2_ kinetics are slowed, oxymyoglobin availability is attenuated [35]. Such a situation of “partial hypoxia” may lead to a decline in PCr concentrations and to an increase in anaerobic glycolysis towards ATP supply. Our data support this idea, as SIT and HIIT elicit similar blood lactate values that are higher compared to CT (Figure 2) with a large effect size (≥ 1.80), despite equal exercise duration. Our outcomes are supported by those obtained in active men and women performing lab-based cycling [36]. A likely mechanism explaining this result is the higher recruitment of type IIx glycolytic muscle fibers required by both interval regimens. Similar findings were observed by Eigendorf et al. [14], who compared three exercise approaches at 50% maximal power output (SIT 6 s work × 24 s rest: 33 min; HIIT 30 s work × 30 s rest: 38 min; CT: 45 min). Furthermore, our protocols showed a lesser blood lactate accumulation than a remarkable number of previous designs that applied SIT or HIIT [11]. Overall, reduced durations of effort, regardless of the exercise modality used (i.e., cycling or running), seem to rely more on oxidative metabolism with less dependence on glycolytic metabolism, attenuating the residual fatigue [12,13,19,34,37].

The pleasure:displeasure and enjoyment experienced during exercise could be a predictor of future engagement in exercise programs [5]. When exercise intensity surpasses the workload associated with the anaerobic threshold, affective valence declines regardless of total work completed [38]. In this regard, some studies have compared the psychological responses to interval training vs. CT matched for internal and external parameters. For example, Jung et al. [39] documented that HIIT (10 bouts of 1 min at ~100% × 1 min at ~20% maximal power output) was more enjoyable and preferable than CT (20 min at 80% maximal power output) and CT (40 min at 40% maximal power output) despite a more aversive response. In contrast, Oliveira et al. [40] observed a lower affective valence in response to HIIT vs. CT at the same average intensity (85% of respiratory compensation point). Similarly, Saanijoki et al. [41] indicated that perceived exertion and arousal was more negative after Wingate-based SIT vs. CT. However, Oliveira et al. [40] and Saanijoki et al. [41] employed relatively high-volume regimens of HIIT (2 min) and SIT (30 s) which induce marked disruptions of homeostasis. Another study that exhibited more positive affective valence did require very short efforts (5 s) [4]. Interestingly, we found that 15 s bouts of HIIT show the most aversive response (Figure 3) and a significant post-effort sensation of fatigue (effect size = 0.84). Despite these differences, no difference in enjoyment was reported, supporting previous data [36,40]. Alternatively, it could be that the PACES scale is cognitive, and affective valence is not, and is more reflective of an more instantaneous perception of exercise. The opponent-process theory states that pleasant affective states are easier to achieve after adverse physical stimulus, as a kind of reward mechanism [42]. This may be linked to the release of chemical modulatory neurotransmitters associated with pleasure and decreased anxiety [40]. Moreover, the intermittent nature of interval training induces a “rebound effect” that generates a better balance of pleasure [39]. However, we did not detect differences in affective valence pre-exercise, given that 62% of the change in psychological variables in response to SIT and HIIT was explained by baseline values [43]. Therefore, it can be argued that the format of interval training protocols implemented in our study (HIIT and SIT) does not harm tolerance or perceptual response. However, chronic studies are necessary to further explore this hypothesis. In this sense, present findings indicate that self-paced HIIT can induce greater physical enjoyment than CT in active young adults [44].

In order to optimize responses to exercise training, it is important to monitor fatigue, fitness, or performance adaptations [17]. Heart rate variability is a non-invasive metric that can be applied daily in large groups to assess the magnitude of physiological recovery using cardiac autonomic balance [17]. It is evident that interval training sessions should be conducted when vagal HRV indicators are high, when they return to baseline, or even when they exceed values from baseline assessments [11]. We observed that lnRMSSD, which represents the vagal influence on autonomic control, is similar over the 24 h after completion of SIT, HIIT, and CT. In fact, differences only were noted for all conditions immediately post-exercise (Figure 4). Although greater anaerobic metabolism occurs during SIT and HIIT, this phenomenon was not reflected in sympathovagal balance. Likewise, no differences were observed in any variable between protocols 6 and 24 h post-exercise, which is attributed to the low-time commitment of these sessions leading to a relatively rapid recovery.

One interesting finding was the lack of difference in CMJ and RSI post-exercise (Figure 5). These tests can be used to assess potential reductions in neuromuscular function and onset of residual fatigue in field conditions. The results suggest that low-volume, intense exercise sessions having distinct stimuli and structure (i.e., “all-out”, intermittent submaximal, and continuous submaximal) do not impair the capacity of subsequent muscular contraction. Similarly, our group reported that a ~12 min session of 5 s sprints did not mitigate subsequent performance in a vertical jump test in healthy active males [19]. Previously, others demonstrated that the small training workload of HIIT and CT did not increase markers of muscle damage and inflammation 48 h post-exercise [16]. Thus, low-volume regimens as completed in the present study may be useful to implement in active adults who choose concurrent training or exercise twice daily, as these bouts do not seem to reduce subsequent neuromuscular function.

Our results from the Hooper index show no significant changes in the general recovery time-course patterns for any regimen. Previously, an association between changes in lnRMSSD and Hooper index has been reported in sub-elite athletes during subsequent days to the competition [45], suggesting that this approach could be an easy way to quantify global psychophysiological stress. Overall, affordable and practical tools such as HRV, jump performance, and psychometric questionnaires could help practitioners and coaches monitor acute and delayed metabolic responses and onset of functional overreaching after different exercise protocols. Additionally, there were no differences from pre- to post-exercise in multiple indicators of sleep quality and daily PA, which is likely due to the low-volume of our regimens. These variables are paramount to consider since they can significantly influence internal and external responses allowing return to homeostasis.

This study has some limitations that must be considered. First, HR during interval exercise may have a faster kinetic response than VO_2_ and other physiological mechanisms, including acidosis; thus, it may not be the most robust marker for monitoring exercise intensity [17]. However, due to the development of wearable technology, HR can be an useful indicator of internal load, since the perception of effort may not be accurate in inactive adults. Second, variables related to the recovery status were only monitored for a single day after exercise, so we do not know how these variables would change if assessed over an extended period. However, our participants regularly engaged in team sports, resistance training, and endurance exercise, which may have conditioned their psychological or physiological responses. Third, our sample was small, physically trained, and included predominantly men, so these findings cannot be generalized to higher volume protocols, women, or inactive populations.

## 5. Conclusions

Our findings suggest that brief bouts of HIIT and CT with a low-time commitment (~14 min) elicit a substantial cardiorespiratory demand ≥ 90 %HR_peak_ that is superior to that induced by SIT. Moreover, our data support those from laboratory-based protocols requiring cycling or running and demonstrate that track-based HIIT consisting of repeated brief efforts can elicit the intense characteristics of interval training while being more accessible to the general population. As a high contribution of anaerobic metabolism attendant with HIIT and SIT could generate more aversive psychological responses, it is advisable to start a training program with a few brief efforts (i.e., 4–6) and then gradually increase bout duration or number. Additionally, it is necessary to examine these training models under unsupervised circumstances, seeking to progress on real-world conditions that are truly scalable in the long-term. Finally, strength and conditioning coaches have to take into account that when endurance exercise sessions are reduced (i.e., < 15 min), regardless of the type of stimulus (SIT, HIIT, or CT), the recovery process can be completed quickly (~6 h).

## Figures and Tables

**Table 1:** Participants’ characteristics.

Variable	
Age (yr)	25.9 ± 5.6
Height (cm)	173.2 ± 7.6
Weight (kg)	66.3 ± 7.3
BMI (kg·m^−2^)	22.1 ± 1.8
Body fat mass (%)	19.0 ± 7.6
Body skeletal muscle mass (%)	38.5 ± 5.8
HR_rest_ (beat·min^−1^)	55 ± 5
HR_peak_ SRT (beat·min^−1^)	200 ± 9
VO_2max_ SRT (mL·kg^−1^·min^−1^)	50.9 ± 5.4

BMI = body mass index; HR_peak_ = peak heart rate; HR_rest_ = rest heart rate; VO_2max_ = maximum oxygen consumption; SRT = shuttle run test. Data are mean ± SD.

**Table 2:** HR response for the training protocols.

Variable	SIT	HIIT	CT
Total exercise duration (min)	13.75	13.75	13.75
Exercise duration (min)	8.75	8.75	8.75
Number of efforts	14	18	1
Work/recovery (s)	5/35	15/15	None
Intensity	all-out	~90 %HR_peak_	~85 %HR_peak_
Distance (m)	428.8 ± 27.4 *	1318.4 ± 112.9 †	1784.5 ± 192.6
HR_max_ (beat·min^−1^)	177 ± 10	185 ± 10 †	182 ± 8
HR_mean_ (beat·min^−1^)	166 ± 10 *	175 ± 8	173 ± 8
HR_min_ (beat·min^−1^)	146 ± 15	148 ± 18	134 ± 17
time ≥ 70 %HR_peak_ (s)	114 ± 100	30 ± 32	18 ± 29
time ≥ 80 %HR_peak_ (s)	384 ± 99	300 ± 141	414 ± 143
time ≥ 90 %HR_peak_ (s)	30 ± 58 ‡	180 ± 141	90 ± 156
HR_end_ (beat·min^−1^)	168 ± 12	182 ± 10†	174 ± 8
HRΔ30s (beat·min^−1^)	14 ± 8	15 ± 5	18 ± 11
HRΔ60s (beat·min^−1^)	27 ± 9	30 ± 10	36 ± 13
HRΔ90s (beat·min^−1^)	36 ± 11 *	49 ± 7	50 ± 11
HRΔ120s (beat·min^−1^)	46 ± 13 ‡	59 ± 6	53 ± 10

SIT = sprint interval training with 5 s efforts; HIIT = high-intensity interval training with 15 s efforts; CT = continuous training; HR = heart rate. Data are mean ± SD. * *p* < 0.05 vs. HIIT and CT. † *p* < 0.05 vs. SIT and CT. ‡ *p* < 0.05 vs. HIIT.

**Table 3:** Incidental PA and sleep for the training protocols.

	SIT	HIIT	CT	SIT	HIIT	CT
Variable	Before 48 h			After 24 h		
INCIDENTAL PA						
Kcal·day^−1^	717.5 ± 185	842.4 ± 300.1	849.7 ± 239.2	1138 ± 351.5	1303.4 ± 418.3	1218.9 ± 458.6
%sedentary domain	74.6 ± 5.5	70.6 ± 9.9	68.1 ± 7.2	69.6 ± 5.7	65.8 ± 6.3	68.3 ± 6.4
%light domain	13.6 ± 3	15.4 ± 4.7	17.3 ± 3.1	15 ± 4.9	16.2 ± 3	15.1 ± 3.7
%MVPA domain	11.8 ± 3	14 ± 5.8	14.6 ± 4.9	15.5 ± 1.2	18 ± 4.5	16.6 ± 3.2
SLEEP						
Efficiency	88.1 ± 5.9	89.2 ± 4.3	90 ± 3.3	89.8 ± 5.1	91.6 ± 4.4	89.6 ± 5.3
TST	365 ± 76.4	373.9 ± 106.2	366.4 ± 68.5	360.1 ± 90.3	317.3 ± 82.2	349.2 ± 85.6
WASO	49.8 ± 26.6	43.9 ± 16.5	38.6 ± 18.9	43 ± 24.3	27.8 ± 11.5	37.7 ± 17.8
Nºawakenings	17.6 ± 6.6	19.5 ± 6.5	16.8 ± 7.8	18.8 ± 11.4	15.1 ± 6	17.7 ± 8.6

SIT = sprint interval training with 5 s efforts; HIIT = high-intensity interval training with 15 s efforts; CT = continuous training; MVPA = moderate vigorous physical activity; WASO = wake after sleep onset; TST = total sleep time. Data are mean ± SD.

## Data Availability

Data pertaining to this research study are available from the corresponding author upon reasonable request.
